# A systematic review of the implementation and impact of asthma protocols

**DOI:** 10.1186/1472-6947-14-82

**Published:** 2014-09-09

**Authors:** Judith W Dexheimer, Elizabeth M Borycki, Kou-Wei Chiu, Kevin B Johnson, Dominik Aronsky

**Affiliations:** 1 Division of Emergency Medicine, Cincinnati Children’s Hospital Medical Center, MLC 2008, 3333 Burnet Avenue, Cincinnati, OH 45229-3039, USA; 2 Division of Biomedical Informatics, Cincinnati Children’s Hospital Medical Center, MLC 2008, 3333 Burnet Avenue, Cincinnati, OH 45229-3039, USA; 3 School of Health Information Sciences, University of Victoria, PO Box 3050 STN CSC, Victoria, BC V8W 3P5, Canada; 4 Department of Biomedical Informatics, Vanderbilt University, 400 Eskind Biomedical Library, 2209 Garland Avenue, Nashville, TN 37232, USA; 5 Department of Emergency Medicine, Vanderbilt University, 400 Eskind Biomedical Library, 2209 Garland Avenue, Nashville, TN 37232, USA

**Keywords:** Review, Asthma, Medical informatics, Systematic review

## Abstract

**Background:**

Asthma is one of the most common childhood illnesses. Guideline-driven clinical care positively affects patient outcomes for care. There are several asthma guidelines and reminder methods for implementation to help integrate them into clinical workflow. Our goal is to determine the most prevalent method of guideline implementation; establish which methods significantly improved clinical care; and identify the factors most commonly associated with a successful and sustainable implementation.

**Methods:**

PUBMED (MEDLINE), OVID CINAHL, ISI Web of Science, and EMBASE.

Study Selection: Studies were included if they evaluated an asthma protocol or prompt, evaluated an intervention, a clinical trial of a protocol implementation, and qualitative studies as part of a protocol intervention. Studies were excluded if they had non-human subjects, were studies on efficacy and effectiveness of drugs, did not include an evaluation component, studied an educational intervention only, or were a case report, survey, editorial, letter to the editor.

**Results:**

From 14,478 abstracts, we included 101 full-text articles in the analysis. The most frequent study design was pre-post, followed by prospective, population based case series or consecutive case series, and randomized trials. Paper-based reminders were the most frequent with fully computerized, then computer generated, and other modalities. No study reported a decrease in health care practitioner performance or declining patient outcomes. The most common primary outcome measure was compliance with provided or prescribing guidelines, key clinical indicators such as patient outcomes or quality of life, and length of stay.

**Conclusions:**

Paper-based implementations are by far the most popular approach to implement a guideline or protocol. The number of publications on asthma protocol reminder systems is increasing. The number of computerized and computer-generated studies is also increasing. Asthma guidelines generally improved patient care and practitioner performance regardless of the implementation method.

## Background

### Asthma disease burden

Asthma is the most common chronic childhood disease in the U.S., affecting 9 million individuals under 18 years of age (12.5%) [1,2]. Approximately 4 million children experience an asthma exacerbation annually resulting in more than 1.8 million emergency department (ED) visits and an estimated 14 million missed school days each year [2,3]. In the U.S., asthma is the third leading cause for hospitalizations among patients <18 years of age [4]. Asthma exacerbations leading to ED encounters and hospitalizations account for >60% of asthma-related costs [5].

### Characteristics of clinical guidelines

Guideline-driven clinical care, in which providers follow evidence-based treatment recommendations for given medical conditions, positively affects patient outcomes for routine clinical care as well as asthma treatment in particular [6-9]. Care providers, payors, federal agencies, healthcare institutions, and patient organizations support the development, implementation, and application of clinical guidelines in order to standardize treatments and quality of care. Consequently, the number of nationally endorsed and locally developed guidelines has grown with 2331 active guidelines found on the US department of Health and Human Services website [10].

Several guidelines exist to support clinicians in providing adequate asthma treatment, including Global Initiative for Asthma Guidelines [11], the British Thoracic Society Guidelines [12], Australian national guidelines [13], and the guideline from the U.S. National Heart Lung and Blood Institute (NHLBI) [14].

There are several reminder methods of implementing guidelines to integrate them into clinical workflow. Reminders can be paper-based, computer-generated or computerized reminders depending on the particular clinic. Reminder methods are defined as follows:

a) Paper-based implementation approaches included the use of paper within the patient’s chart in the form of stickers, tags, or sheets of paper and patients were identified manually by office staff.

b) Computer-generated implementations included the application of computerized algorithms to identify eligible patients, but the reminder or protocol was printed out and placed in the patient chart or given to the clinician during the visit.

c) Computerized reminders included prompts that were entirely electronic, i.e., computerized algorithms identified eligible patients, and prompts were provided upon access to the electronic clinical information system [15].

However, time and guideline initiation can limit the integration of guidelines in the daily routine of practicing clinicians, [6] and many implementation efforts have been shown little effect [16]. We believe that asthma guidelines would be used more frequently if clinicians were aware of the best published implementation methods. The objective of our systematic literature review was to determine the most prevalent method of guideline implementation (paper, computer-generated, or computerized), as reported in the literature; establish which methods significantly improved clinical care; and identify the factors most commonly associated with a successful and sustainable asthma guideline implementation.

## Methods

### Literature search

We conducted a systematic literature review to identify articles that studied the impact of implementing paper-based and computerized asthma care protocols and guidelines in any clinical setting, including treatment protocols, clinical pathways, and guidelines. We did not create a central review protocol and followed PRISMA guidelines; however we were unable to perform meta-analysis [17]. Studies were eligible for inclusion if they examined asthma protocol implementation for clinicians or patients, evaluated an intervention and not just the design, were a clinical trial of a protocol implementation, and qualitative studies as part of a protocol intervention. Studies were excluded if they enrolled non-human subjects, studied the efficacy and effectiveness of drugs, lacked an evaluation component, tested no intervention, studied a clinician or patient educational intervention only, or were a case report, survey, editorial, letter to the editor, or non-English language report.

We searched the electronic literature databases PUBMED® (MEDLINE®) [18], OVID CINAHL® [19], ISI Web of Science™ [20], and EMBASE ® [19] from their respective inception to December 2010. In MEDLINE, all search terms were defined as keywords and Medical Subject Headings (MeSH®) unless otherwise noted; in the remaining databases, the search terms were defined only as keywords. The search strategy was based on the concept “asthma” combined with concepts representing any kind of asthma protocol implementation. Search terms included ‘asthma’ and any combination of the terms ‘checklist’, ‘reminder systems’, ‘reminder’, ‘guideline’, ‘pathway’, ‘flow diagram’, ‘guideline adherence’, ‘protocol’, ‘care map’, ‘computer’, ‘medical informatics’, ‘informatics’ and relevant plurals. The exact PubMed query is shown below:

asthma AND (medical informatics OR computers OR computer OR informatics OR checklist OR checklists OR reminder systems OR reminder OR guideline OR pathway OR pathways OR “flow diagram” OR guidelines OR guideline adherence OR protocol OR protocols OR “care map” OR “care maps”)

### Review of identified studies

The title and abstract of all articles identified using the keyword searches were retrieved and reviewed by two of three independent reviewers (JWD, KWC, DA). Disagreements between two reviewers were resolved by consensus among all three participating reviewers. The bibliographies of identified review articles were examined and additional relevant studies were included. All included studies were examined for redundancy (e.g., findings of one study reported in two different reports) and duplicate results were removed. The full text of included articles was obtained and two reviewers (JWD, DA) screened the articles independently for inclusion. Disagreements were resolved by consensus. Data were abstracted by one reviewer (JWD) into a central database. To obtain a better understanding of implementation approaches, studies were further categorized as “paper-based,” “computer-generated,” or “computerized [15]”.

### Analysis

We collected basic demographic data from each study including reminder type [15], setting, study design, randomization, patient and clinician populations, setting, the centers (multicenter or single center) and factors described below. We looked at all included studies to determine similar characteristics associated with implementing guidelines, study design, and study scoring. We assessed study quality following the methodology of Wang et al., which grades study design on a 5-point scale with Level 1 studies being the most scientifically rigorous and Level 5 studies having a more lenient study design [21]. The study levels were adapted as follows:

1. Level 1 studies were primary prospective studies, case–control groups of consecutive or random patients.

2. Level 2 studies were similar to Level 1 but with a smaller sample size.

3. Level 3 studies were retrospective studies, non-random designs, or non-consecutive comparison groups.

4. Level 4 studies had a reference standard or convenience sample of patients who have the target illness.

5. Level 5 studies were comparisons of clinical findings with a reference or convenience of unknown or uncertain validity.

The effects of the implementation on the performance were graded based on Hunt et al. [22]. The intervention effects on health care practitioner performance and patient outcomes were examined. Studies were classified to have no change, a decreased change, or an increased change. A positive improvement in reported patient outcomes was an increased change; a negative effect such as a decrease in the number of action plans given after implementation were considered a decreased change. A positive improvement in reported measure of health care practitioner performance such as guideline compliance or increased charting was considered an increased change in performance; a reported decrease in the measurement was considered a decreased change.

We assessed success factors following the methodology of Kawamoto et al. [23]. The success factors for each study were determined from the article’s text. If the success factors of the implementation could not be determined or were not present in the article, we contacted the authors. The success factors were designed from and are intended to be applied to clinical decision support systems. We applied the factors to all three study types. The success factors are listed in Table 1. When the prompt information was unavailable, the study authors were contacted in an attempt to obtain it.

**Table 1:** Intervention effects from Hunt et al

	**No change**	**Decreased**	**Increased**
Significant Effect on Health Care Practitioner Performance	32	0	66
Significant Effect on Patient Outcome	37	0	64

Agreement among reviewers to consider articles based on title and abstract was high (0.972 to 0.996), as determined by Yule’s Q [24].

Yule'sQ=OddsRatio−1OddsRatio+1

## Results

**Figure 1:**
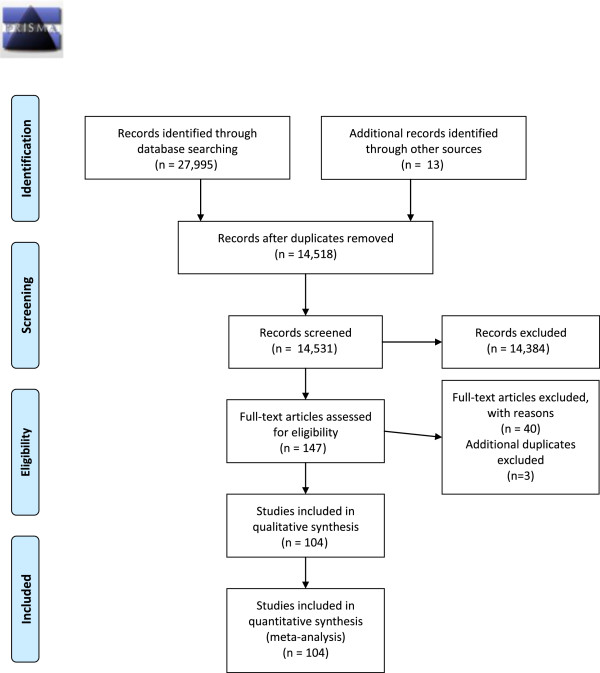
PRISMA 2009 flow diagram.

The literature searches resulted in 27,995 abstracts during the search period (Figure 1). After excluding 13,477 duplicates 14,384 articles were further excluded based on a review of the title and abstract, leaving 134 articles for further consideration. We retrieved the full text of the 134 articles and added 13 articles for full-text review that were identified from the bibliographies of the 104 full text studies. From the 147 articles we excluded 39 studies not meeting inclusion criteria based on the full-text information.

The 43 articles we removed from the study set included resource utilization studies (2 articles), implementation/design/development/system descriptions without evaluations (10 articles), drug trial publications (7), surveys (7), no intervention, descriptive or protocol descriptions (6), no guideline implementation (5), reviews (1), overviews of asthma (1), studies of education-only interventions (1), simulation studies (1), abstracts (1), and one study that only provided data on the efficacy of guidelines (not an intervention). We included 104 full-text articles for evaluation (Figure 1). We extracted data from 101 articles. Two sets of articles contained the same intervention and therefore only one was included in the analysis, these were 3 articles [25-27] and 2 articles [28,29].

**Figure 2:**
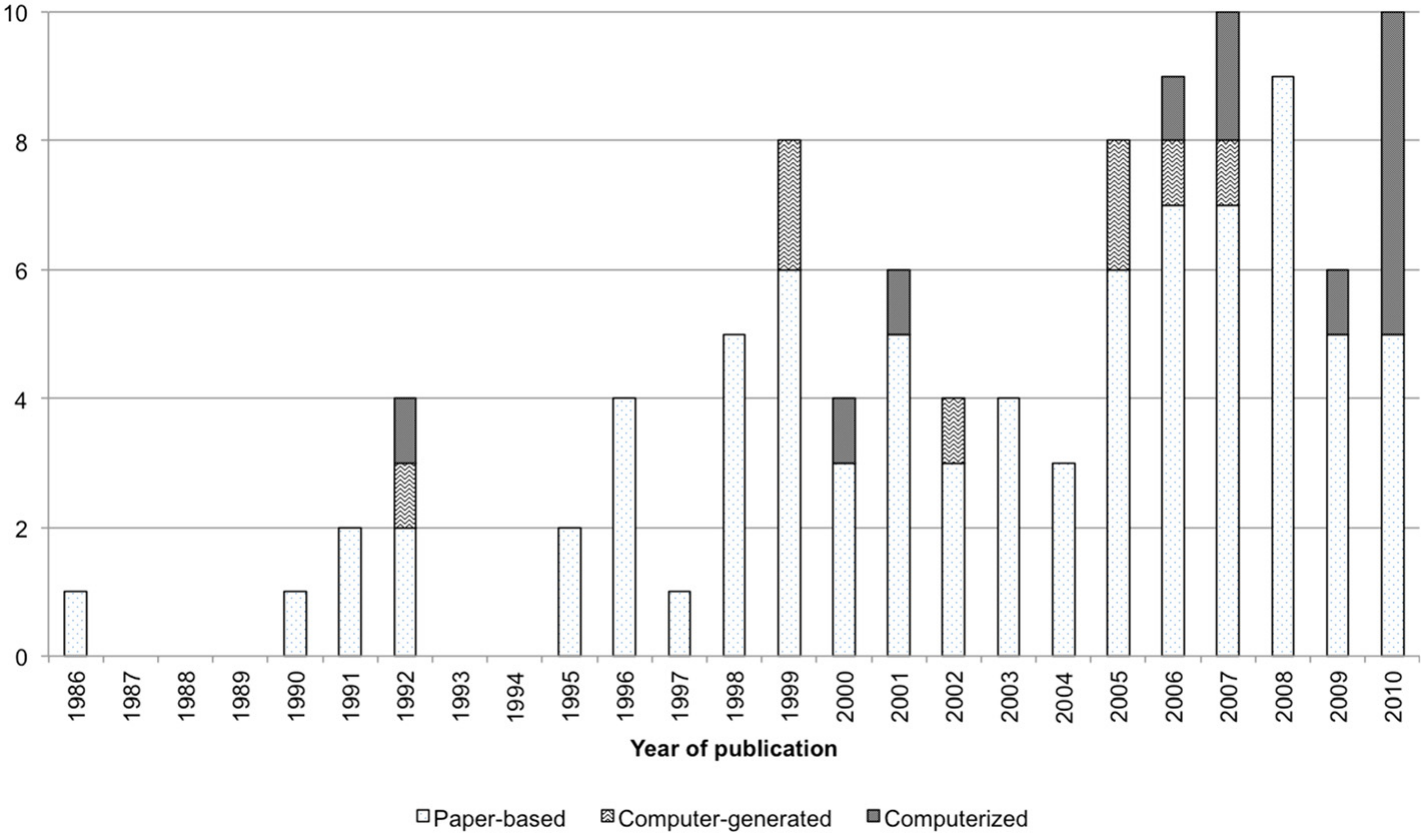
Number of publications per publication year based on intervention classification.

We identified the guideline implementation method, study setting, study design, randomization, patient population, clinician population, setting, and study center count for all 101 articles (Table 2). Study publication years ranged from 1986 to 2010, with a peak of 10 studies in 2010 (Figure 2). Of the studies that reported a guideline 75 used site-specific guidelines, 66 used national guidelines, and 1 used another protocol. Forty-eight studies adapted a national guideline to be site-specific. Study periods ranged from 2 months to 114 months. Patient follow-up ranged from half a day to 730 days. In 59 studies the physician was the clinician studied, nurses were studied in 25 studies, respiratory therapists in 8, and other clinicians in 3 studies. Of the studies that mentioned the clinician population, the range of participants was 8 to 377. Of studies that mentioned the total patient population size, the range of participants was 18 to 27,725.

**Table 2:** Demographics of included studies

**Ref**	**Author**	**Year**	**Reminder type**	**Setting**	**Study Design**	**Randomized**	**Patient Population**	**Clinician Population**	**Setting**	**Centers**
[30]	Abisheganaden J	2001	Pa	Acad	Retro	0	Adult	MD	IN	Single
[31]	Abisheganaden J	1998	Pa	Other	Descrip	0	Adult	O	ED	Single
[32]	Ables A	2002	Pa	Acad	Pro	0	Adult	MD	OUT	Single
[33]	Akerman M	1999	Pa	nonAcad	Pro	0	Adult	MD	ED	Single
[24]	Alamoudi O	2002	Pa	Acad	Pro	0	Adult	MD	OUT	Single
[34]	Baddar S	2006	Pa	Acad	Pro	0	Adult	MD	OUT	Multi
[35]	Bailey R	1998	Pa	Acad	Pro	0	Adult	MD	IN	Single
[36]	Baker R	2003	Pa	nonAcad	Pro	1	Adult	MD	OUT	Multi
[37]	Bell LM	2010	CP	nonAcad	Pro	1	PED	MD, RN	OUT	Multi
[38]	Boskabady MH	2008	Pa	Acad	Pro	0	Adult	O	OUT	Single
[39]	Callahan C	2003	Pa	Acad	Pro	0	PED	MD	OUT	Single
[40]	Cerci Neto AC	2008	Pa	nonAcad	Retro	0	Adult, PED	MD, RN	IN	Multi
[41]	Chamnan	2010	Pa	nonAcad	Other	0	Adult	O	Out	Single
[42]	Chan D	2007	CP	nonAcad	Pro	1	PED	O	OUT	Single
[43]	Chee C	1996	Pa	nonAcad	Retro	0	Adult	O	IN	Single
[44]	Cho SH	2010	CP	nonAcad	Pro	0	Adult	MD	OUT	Multi
[45]	Chouaid C	2004	Pa	Acad	Retro	0	Adult	O	ED	Single
[46]	Cloutier M	2006	Pa	nonAcad	Retro	1	PED	MD	OUT	Multi
[47]	Cloutier M	2005	Pa	Acad	Retro	0	PED	MD	OUT	Multi
[48]	Colice G	2005	CG	Acad	Pro	0	Adult	RT	IN	Single
[49]	Cunningham S	2008	Pa	Acad	Pro	1	PED	O	ED	Single
[50]	Dalcin P	2007	CG	Acad	Pro	0	Adult	O	ED	Single
[51]	Davies B	2008	Pa	nonAcad	Pro	0	Adult	RN	OUT, ED	Multi
[52]	Davis AM	2010	CP	Acad	Retro	0	Adult	MD	OUT	Single
[25-27]	Doherty S	2007	Pa	nonAcad	Retro	0	Adult	O	ED	Multi
[53]	Duke T	1991	Pa	Acad	Pro	0	PED	MD	ED	Single
[54]	Eccles M	2002	CG	nonAcad	Retro	1	Adult	MD	OUT	Multi
[55]	Emond S	1999	Pa	Acad	Retro	0	Adult	O	ED	Single
[56]	Feder G	1995	Pa	nonAcad	Pro	1	Adult	O	OUT	Multi
[57]	Fifield J	2010	CP	nonAcad	Pro	0	PED	O	OUT	Multi
[58]	Gentile N	2003	Pa	Acad	Retro	0	Adult	MD	ED	Single
[59]	Gibson P	1996	Pa	nonAcad	Descrip	0	Adult	MD	IN	Single
[60]	Gildenhuys J	2009	Pa	Acad	Retro	0	PED	O	ED	Single
[61]	Goh AEN	2010	Pa	nonAcad	Retro	0	PED	O	IN, ED	Single
[62]	Goldberg R	1998	Pa	Other	Pro	0	Adult	RN	OUT	Single
[63]	Guarnaccia S	2007	Pa	Acad	Descrip	0	PED	MD	OUT	Multi
[64]	Hagmolen of ten Have W	2008	Pa	nonAcad	Pro	1	PED	MD	OUT	Multi
[65]	Halterman JS	2006	Pa	Acad	Pro	1	PED	O	OUT	Multi
[66]	Heaney L	2003	Pa	nonAcad	Pro	0	Adult	MD	OUT	Multi
[67]	Jans M	2001	Pa	nonAcad	Descrip	0	PED	O	OUT	Multi
[68]	Jans MP	1998	Pa	nonAcad	Descrip	0	Adult	MD	OUT	Multi
[69]	Joe R	1992	Pa	Acad	Descrip	0	Adult	MD	ED	Single
[70]	Johnson K	2000	Pa	Acad	Pro	1	PED	RN	IN	Single
[71]	Jones CA	2007	Pa	Acad	Other	0	PED	O	OUT, Other	Multi
[72]	Kelly A	2007	Other	nonAcad	Descrip	0	Adult, PED	O	ED	Multi
[73]	Kelly C	2000	Pa	Acad	Retro	1	PED	MD	IN	Single
[7]	Kuilboer M	2006	CP	nonAcad	Pro	1	Adult	MD	OUT	Multi
[74]	Kwan-Gett T	1997	Pa	Acad	Retro	0	PED	RN	IN	Single
[75]	Kwok R	2009	CP	nonAcad	Retro	0	Adult	O	ED	Single
[76]	Lehman HK	2006	Pa	nonAcad	Pro	0	PED	MD	OUT	Multi
[77]	Lesho E	2005	Pa	nonAcad	Pro	0	Adult	MD	OUT	Multi
[78]	Lierl M	1999	Pa	nonAcad	Pro	0	PED	RT	IN	Single
[79]	Lim T	2000	Pa	Acad	Pro	0	Adult	MD	IN	Single
[80]	Lougheed MD	2009	Pa	Acad	Retro	0	Adult	O	ED	Multi
[81]	Lukacs S	2002	Pa	Acad	Pro	0	PED	O	OUT	Multi
[82]	Maa SA	2010	CP	nonAcad	Pro	0	PED	O	Other	Single
[83]	Mackey D	2007	Pa	Acad	Pro	0	Adult	MD	ED	Single
[84]	Martens JD	2007	CP	nonAcad	Pro	1	None	MD	OUT	Multi
[85]	Martin E	2001	Pa	nonAcad	Retro	0	PED	MD	OUT	Multi
[86]	Massie J	2004	Pa	Acad	Descrip	0	PED	O	ED	Single
[87]	Mccowan C	2001	CP	nonAcad	Descrip	1	Adult	MD	OUT	Multi
[88]	McDowell K	1998	Pa	Acad	Pro	0	PED	MD	IN	Single
[89]	McFadden E	1995	Pa	Acad	Pro	0	Adult	MD	ED	Single
[90]	Mitchell E	2005	Pa	nonAcad	Pro	1	PED	MD	OUT	Multi
[91]	Nelson K	2009	Pa	Acad	Retro	0	PED	RN	Other	Single
[92]	Newcomb P	2006	Pa	Acad	Pro	0	PED	RN	OUT	Single
[93]	Norton S	2007	Pa	Acad	Pro	0	PED	MD	ED	Single
[94]	Patel P	2004	Pa	nonAcad	Retro	0	Adult	MD	OUT	Multi
[95]	Porter S	2006	CG	Acad	Pro	0	PED	MD	ED	Single
[96]	Press S	1991	Pa	Acad	Pro	0	PED	O	ED	Single
[97]	Qazi K	2010	Pa	nonAcad	Pro	0	PED	RN	ED	Single
[98]	Quint DM	2009	Pa	Acad	Pro	1	PED	O	ED	Single
[99]	Renzi P	2006	Pa	nonAcad	Pro	1	Adult	MD	OUT	Multi
[100]	Robinson S	1996	Pa	Acad	Pro	0	Adult	O	ED	Single
[101]	Rowe BH	2008	Pa	Acad	Retro	0	Adult	MD, RT	ED	Single
[102]	Ruoff G	2002	Pa	nonAcad	Retro	1	Adult	MD	OUT	Single
[103]	Schneider A	2008	Pa	nonAcad	Pro	1	Adult	MD	OUT	Multi
[104]	Schneider S	1986	Pa	Acad	Retro	0	Adult	MD	ED	Single
[105]	Shelledy D	2005	Pa	Acad	Pro	0	PED	RT	IN	Single
[106]	Sherman J	1997	Pa	Acad	Descrip	0	PED	MD	Other	Multi
[9]	Shiffman R	2000	CP	nonAcad	Pro	1	PED	MD	OUT	Multi
[107]	Stead L	1999	Pa	Acad	Retro	0	Adult	O	ED	Single
[108]	Stell I	1996	Pa	nonAcad	Retro	0	Adult	MD	ED	Single
[109]	Steurer-Stey C	2005	Pa	Acad	Pro	0	Adult	MD	ED	Single
[110]	Stormon M	1999	Pa	Acad	Pro	1	PED	O	IN	Single
[111]	Sucov A	2000	Pa	Acad	Pro	0	Adult	MD	ED	Single
[112]	Suh D	2001	Pa	Acad	Retro	0	Adult	MD	IN	Single
[113]	Sulaiman NB	2010	Pa	nonAcad	Pro	1	PED	MD	OUT	Multi
[114]	Suzuki T	2010	Pa	Acad	Retro	0	Adult	MD	OUT	Single
[115]	Szilagyi P	1992	CP	Acad	Pro	1	PED	MD	OUT	Single
[116]	Thomas K	1999	CG	Other	Descrip	1	PED	MD	Other	Single
[117]	Tierney W	2005	CG	Acad	Pro	1	Adult	O	OUT	Single
[28,29]	To T	2008	Pa	nonAcad	Pro	0	Adult, PED	O	OUT	Multi
[118]	Touzin K	2008	Pa	Acad	Retro, Descrip	0	PED	MD	ED	Single
[119]	Town I	1990	Pa	Acad	Retro	0	Adult	MD	ED	Single
[120]	van de Meer V	2010	Other	nonAcad	Pro	1	Adult	O	OUT	Multi
[121]	Vandeleur M	2009	Pa	Acad	Retro	0	PED	MD, RN	IN	Single
[122]	Wazeka A	2001	Pa	Acad	Retro	0	PED	O	IN	Single
[123]	Webb L	1992	Pa	Acad	Pro	0	PED	O	IN	Single
[124]	Welsh K	1999	CG	Acad	Retro	0	PED	MD	IN	Single
[125]	Wright J	2003	Pa	nonAcad	Pro	0	Adult	MD	OUT	Multi

Table 1 key: CG – computer generated, Pa – paper-based, CP – computerized. Acad –academic setting, nonAcad – non academic setting, Pro – prospective, Retro – retrospective, Descrip – Descriptive, PED – pediatric, O – other, MD – physician, RN – nurse, RT – respiratory therapist, IN inpatient, OUT – outpatient, ED – emergency department, Multi – multi-center trial.

Studies were performed in the United States (48 studies), the United Kingdom (10 studies), Canada (9 studies), Australia (8 studies), the Netherlands (6 studies), Singapore (5 studies), New Zealand (2 studies), Brazil (2 studies), Saudi Arabia (2 studies), Germany (2 studies), and 1 study each in France, Oman, Switzerland, Italy, Iran, Japan, Taiwan, Korea, Thailand, and the United Arab Emirates.

The most frequent study designs included a pre-post design (61 studies), followed by 56 studies that applied a prospective design, 27 population based case series, 23 consecutive case series, 13 randomized trials, 15 non-blinded trials, 16 nonconsecutive case series, 5 double-blinded trials, and 6 best-case series. Studies could be classified as having more than one design element. Six studies were descriptive and one looked at quality improvement. Most studies were performed at academic institutions (57 studies) with 42 studies performed at non-academic institutions and 3 did not describe the setting. Studies looked at outpatients most frequently (50 studies), followed by the emergency department (39 studies) and inpatients (20 studies), with 7 studies looking at patients in other settings (e.g., the home). Some studies involve multiple settings. Most studies were performed in a single center (64 studies) versus a multi-center environment (38 studies).

Reminders consisted of paper-based (82 studies), computer generated (8 studies), fully computerized (12 studies), and other modalities (10 studies). The interventions were protocol-based (61 studies), treatment-based (53 studies), focused on the continuity of care (17 studies), scoring based (19 studies), and included an educational component (48 studies). Fifty studies reported or described using an asthma scoring metric that was applied to guide treatment decisions. Seventy-three studies listed some or all of the medications suggested for use in asthma management. Forty-two studies included clinician education and 30 studies included patient education (e.g., inhaler technique, asthma education and teaching). If the intervention method was described, 67 described measuring protocol adherence including chart review, severity scoring, checking orders, and the use of the physical protocol. Ten described work-flow interventions, and 2 looked at the timing of care during the patient’s visit.

The effects of the intervention are shown in, Table 2. No study reported a decrease in health care practitioner performance or declining patient outcomes. 66 (63%) studies improved health care practitioner performance and 32 (31%) studies had no change in performance. 34 (33%) studies increased or improved patient outcomes and 37 (36%) resulted without affecting a change in outcomes.

**Figure 3:**
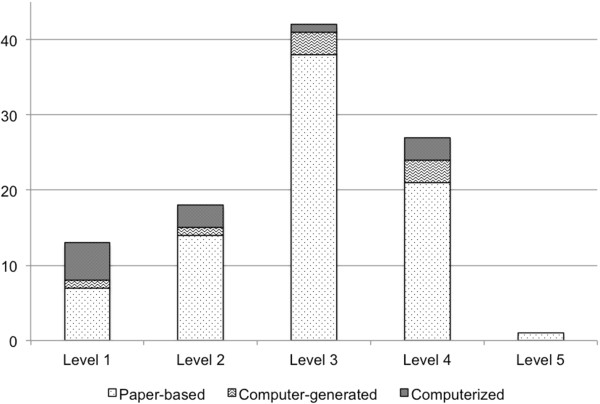
Study quality based on Hunt et al. by intervention classification.

Among the 12 computerized studies, 5 studies with no change in the health care practitioner performance, 7 improved performance. There were 3 studies with no change in the patient outcomes and 9 studies that improved patient outcomes. Among the 8 computer-generated studies 4 resulted in no change in the health care practitioner performance, 4 improved performance. There were 5 studies with no change in the patient outcomes and 3 studies that improved patient outcomes. Paper-based studies had 24 studies with no change in the health care practitioner performance, 56 improved performance. There were 31 studies with no change in the patient outcomes and 51 studies that improved patient outcomes.Study quality is shown in Figure 3. Most studies (41%) were assessed as level 3 quality studies, i.e., retrospective studies, non-random designs, or non-consecutive comparison groups.

The success factors for each study are in Table 3. The number of success factors implemented ranged from 0 to 15, from a maximum of 22 possible. Computerized studies implemented an average of 7.1 success factors (range: 2 to 15). Computer-generated studies implemented an average of 5.7 success factors (range: 3 to 11); and paper-based studies implemented an average of 3.7 success factors (range: 0 to 12). The paper-based implementation most often had a computer help to generate the decision support, the computer-generated and computerized implementations had clear and intuitive interfaces or prompts.

**Table 3:** Success factors

**Success factors**	**Paper-based**	**Computer-generated**	**Computerized**
Accompanied by conventional education	13	5	5
Clear and intuitive user interface with prominent display of advice	3	6	11
System developed through iterative refinement process	4	1	4
Local user involvement in development process	13	3	3
Active involvement of local opinion leaders	1	3	0
Assessments and recommendations are accurate	2	4	5
Saves clinicians time or requires minimal time to use	2	2	1
Provision of decision support results to patients as well as providers	12	3	7
No need for additional clinician data entry	6	2	3
Provision of recommendation, not just an assessment	5	2	4
Accompanied by periodic performance feedback	4	0	2
Integration with charting or order entry system to support workflow integration	31	3	3
Alignment of decision support objectives with organizational priorities and with the beliefs and financial interests of individual clinicians	18	1	3
Promotion of action rather than inaction	12	1	3
Justification of decision support via provision of reasoning	33	0	4
Automatic provision of decision support as part of clinician workflow	0	2	2
Justification of decision support via provision of research evidence	16	1	4
Use of a computer to generate the decision support	38	4	8
Provision of decision support at time and location of decision making	26	0	4
Recommendations executed by noting agreement	28	1	6
Request documentation of the reason for not following recommendations	10	1	1
System is fast	30	1	2

The most common primary outcome measure was compliance with provided guidelines or prescribing guidelines (32 studies), key clinical indicators such as patient outcomes or quality of life were used in 20 studies, and hospital or emergency length of stay in 19 studies. Admission was used as a primary outcome in 8 studies and medication use was looked at in 8 studies including the use of a spacer, timing of medication administration, use of oxygen. Relapse to either the inpatient or emergency department were used in 4 studies; and educational outcomes were used in 2 studies. The administration of an action plan, filling prescriptions, quality improvement, documentation of severity, ED visits, and cost were looked at as primary outcomes in only one study each. One qualitative study was included.

Of the 16 studies that reported a percentage of patients going home on take-home medications either beta-agonists or inhaled corticosteroids, the mean initial reported value was 57% (range: 0.53%, 92%) with a mean final reported value of 69% (range: 14%, 100%). Of the 18 studies that reported the percentage of patients with an asthma action plan or asthma care plan, the mean initial reported value was 20% (range: 0%, 62%) with a mean final reported value of 46% (range: 7%, 100%). Studies (49) that looked at admissions rates between groups reported an initial mean value of 11% (range: 0%, 55%) with a mean final reported value of 9% (range: 0%, 37%) but was highly variable based on selected population. The 38 studies that looked at ED visit rates between groups reported an initial mean value of 9% (range: 0%, 47%) with a mean final reported value of 8% (range: 0%, 46%) also variable by population chosen.

## Discussion

Paper-based implementations are by far the most prevalent method to implement a guideline or protocol. All of the methods implemented either improved clinical care or had no change. Of those that improved patient care, 94 were paper-based, 9 were computerized and only 3 were computer-generated. The paper-based implementation was the most likely to report improving patient care. Of the studies that reported improving patient care, they reported an average of 4.5 success factors with “Clear and intuitive user interface with prominent display of advice” (50%), “Active involvement of local opinion leaders” (41%), and “Local user involvement in development process”(41%) being the most common success factors reported, and 52 (83%) of them also improved practitioner performance. They were most often prospective (59%) and pre-post (63%) study designs. These characteristics are reported as our “best” implementation methods since they improved patient care. Due to the disparate nature of the results across manuscripts, we did not perform a meta-analysis but presented the descriptive data in aggregate form.

Clinical decision support research is difficult to perform. Alerting methodologies and their effectiveness have been studied in the literature but are frequently limited in scope in terms of time and conditions [126-129]. The results suggest that reminder systems are effective at changing behavior and improving care, and they are more successful when designed for a specific environment [127]. This individualized design and the necessary study design demands, help to make clinical decision support more difficult to evaluate homogenously.

The double-blinded randomized controlled trial is considered the gold-standard for study design but it is difficult to implement any kind of reminder system that could be effectively blinded and randomized. While blinding is frequently difficult, decision support implementations can be blinded if the interventions occur at different locations or for different providers. Randomized controlled trials are not well-presented in the informatics literature [130], and many potential issues exist in implementation research including issues such as randomization (e.g. by patient, physician, day, clinic) and outcome measures (e.g. informatics-centric or patient outcomes centric). Failure to consider clinical workflow when implementing reminder systems has impeded guideline adoption and workflow issues can be barriers to adoption [131,132].

Pediatric and adult populations are studied equally. As a chronic condition outpatient studies were most frequent followed by ED-based studies and finally inpatient studies. Few studies reported randomization and a pre-post design was most common. Seventy percent of the studies had a level 3 or higher. The studies were designed optimally for the disparate locations, settings, and factors that needed to be considered. We excluded studies looking at just an educational component for either clinicians or patients because these covered general asthma and guideline knowledge, not implementation or adherence.

No interventions reported decreasing the quality of clinician care or patient care. “No change” in care or an improvement in care or performance was reported in all published studies. This may be due to negative studies not being published. Because of the disparate outcomes measures used, a single characteristic could not be determined to decide which implementation methodology was best or most-effective. Choosing the best implementation method from paper-based, computerized, and computer generated is a situationally dependent task and medical record and workflow considerations for specific settings should be taken into account.

The computerized studies had no change in clinician performance in 42% of the interventions; this may be due to the prompts not being integrated into the clinician’s workflow. The computerized studies mostly reported improving patient outcomes (75%) and having no change on patient outcomes. The computer-generated studies were evenly split on having no change in practitioner performance and improving performance but had 62% of the studies report no change in patient outcomes. The paper-based studies had 70% reporting an improvement in clinician performance and a 62% improvement in the patient outcomes. There were more paper-based than computer-based studies, but paper can be an effective way to implement a protocol reminder. However, as hospitals increase their use of computerized decision support and electronic medical records, it is likely that the efficacy of computer-based protocol implementations will also improve.

Many studies did not implement or report many success factors [33]. These success factors were created for computerized decision support implementations so they may not be as valuable a scoring tool for the paper-based studies. We applied them to the paper-based and computer-generated studies as best as possible (e.g., a paper-based form with check boxes would have required minimal time to use compared to a paper-based form that required writing out entirely new orders by hand). Automatically prompting providers increases adherence to recommendations [133], however in a newer systematic review, effective decision support is still provided to both the patients and physicians and is lower for electronic systems [134]. The benefits of decision support still remain small [135].

The analysis is limited by what results were reported in the manuscripts. Although an attempt was made to contact the corresponding authors, some manuscripts were 20 years old or more and details about the exact intervention may have been lost. Because we only included published manuscripts, a publication bias may exist where studies with positive results are more likely to be published. Given the tendency to publish and emphasize favorable outcomes, decision support systems have the potential to increase adverse outcomes however, these are rarely reported [136].

The outcomes varied from each study and were too disparate to combine. In conclusion, asthma guidelines generally improved patient care and practitioner performance regardless of the implementation method.

## Conclusion

The number of publications on asthma protocol reminder systems is increasing. The number of computerized and computer-generated studies is also increasing. There appears to be a moderate increase towards use of information technology in guideline implementation and will probably continue to rise as electronic health records become more widespread. Asthma guidelines improved patient care and practitioner performance regardless of the implementation method.

## Abbreviations

ED: Emergency department; NHLBI: National heart lung and blood institute.

## Competing interests

The authors declare that they have no competing interests.

## Authors’ contributions

All authors contributed materially to the production of this manuscript. JD participated in the design, acquisition of data, drafting of the manuscript, critical revision, and technical, and material support. EB was involved with drafting of the manuscript and critical revisions. KC participated in the design, article review, and revisions. KJ was involved with the conceptual design and revisions. DA participated in article review, conception, design, and critical revisions. All authors read and approved the final manuscript.

## Pre-publication history

The pre-publication history for this paper can be accessed here:

http://www.biomedcentral.com/1472-6947/14/82/prepub
